# The Australian preventive health agenda: what will this mean for workforce development?

**DOI:** 10.1186/1743-8462-6-14

**Published:** 2009-05-22

**Authors:** Kathleen C Lilley, Donald E Stewart

**Affiliations:** 1 School of Public Health, Griffith University South Bank campus, PO Box 3370 South Brisbane, QLD 4101, Australia; 2 School of Public Health, Griffith University, Logan campus, Meadowbrook, QLD, Australia

## Abstract

The formation of the National Health and Hospitals Reform Commission (NHHRC) and the National Preventative Task Force in 2008, demonstrate a renewed Australian Government commitment to health reform. The re-focus on prevention, bringing it to the centre of health care has significant implications for health service delivery in the primary health care setting, supportive organisational structures and continuing professional development for the existing clinical and public health workforce. It is an opportune time, therefore, to consider new approaches to workforce development aligned to health policy reform. Regardless of the actual recommendations from the NHHRC in June 2009, there will be an emphasis on performance improvements which are accountable and aligned to new preventive health policy, organisational priorites and anticipated improved health outcomes.

To achieve this objective there will be a need for the existing population health workforce, primary health care and non-government sectors to increase their knowledge and understanding of prevention, promotion and protection theory and practice within new organisational frameworks and linked to the community. This shift needs to be part of a national health services research agenda, infrastructure and funding which is supportive of quality continuing professional development.

This paper discusses policy and practice issues related to workforce development as part of an integrated response to the preventive agenda.

## Background

The 2008 National Health and Hospitals Reform Commission (NHHRC) reflects a shift in public policy and Australian Government commitment to health system reform [1]. The eight areas of performance improvement identified in the Commission's Terms of Reference demonstrate a renewed national commitment to a prevention agenda perhaps mirroring the former Hospitals and Health Services Commission Act gazetted by the Whitlam Government in 1974[2].

The areas of performance improvement identified in the Terms of Reference are [3]:

1. reduce inefficiencies generated by cost-shifting, blame-shifting and buck-passing;

2. better integrate and coordinate care across all aspects of the health sector, particularly between primary care and hospital services around key measurable outputs for health;

3. bring a greater focus on prevention to the health system;

4. better integrate acute services and aged care services, and improve the transition between hospital and aged care;

5. improve frontline care to better promote healthy lifestyles and prevent and intervene early in chronic illness;

6. improve the provision of health services in rural areas;

7. improve Indigenous health outcomes; and

8. provide a well qualified and sustainable health workforce into the future.

The NHHRC will report on its long-term plan to achieve sustainable improvements in the Australian health system by June 2009.

The interpretation of the NHHRC terms of reference will probably be viewed quite differently across health sectors. Public health practitioners and academics could argue that they have the conceptual capacity and methodologies to contribute to a number of these performance areas. If this is true, it would be opportune for the sector to ensure that the terms are considered in a broad context. This context should include new partnerships and models for primary health care beyond the existing limits of chronic disease management [4,5] which is commonly seen as the public health sphere of activity. Alternative models for primary health care have been proposed but have not been expanded to identify human resource development plans necessary to support the new policy implications [6,7]. Such models respond to Starfield's [8] arguments that Australia's primary health care sector falls behind other countries in prevention.

While the Australian health system is undergoing this reform process, it is timely to consider potential opportunities for more integrated approaches for human resource development aligned to health policy reform. The first comprehensive Australian health workforce policy document was released in 2004 [9]. While the National Health Workforce Strategic Framework was comprehensive, its implementation plan is not aligned to the prevention agenda or the emerging workforce and education challenges associated with the shift in policy. The National Health Workforce Taskforce was established in 2007 [10] and has recently called for submissions for a National Health Workforce Collaboration [11]; a three-year workforce collaboration research project. While these initiatives are positive for workforce development they do not yet reflect the level of sustainability required for integrated long-term workforce solutions.

To achieve performance improvements in prevention, there will be an urgent requirement for the existing population health workforce, primary health care and non-government sectors to increase their knowledge and understanding of prevention, promotion and protection theory and practice, within new organisational development frameworks. New multi-disciplinary models of care, community engagement and organisational accountability will require new workplace competencies, some of which maybe generic and others specific to particular organisations. Adequate funding for human resource development and new models of continuing professional development will be required to enable collaboration and working to a shared agenda.

This paper discusses policy and practice issues related to human resources for health in context with organisational and workforce development. It argues that to achieve health reform aligned to the preventive agenda; policy initiatives must be aligned to organisational and workforce development through collaboration, leadership, infrastructure, an aligned research agenda and sustainable resourcing.

### Human resources for health

Many authors have recognised the contribution of appropriate and skilled human resources to the success of health system performance [12-15] and [16]. These authors also identify a number of issues acting as barriers to a more coordinated approach to workforce development linked to health policy and organisational goals. Buchan [12] notes that while the evidence base for finance and stewardship issues related to health reform have been investigated, there is limited evidence related to human resources for health. Clinical outcomes are intensely scrutinised, but the contribution of human resource management linked to health system performance and outcomes is limited. He argues that the right fit for human resource policy and management is integral to health system performance and that any interventions targeted at organisational performance should be in context with the organisational priorities. The historical absence of health services research in comparison to biomedical research was noted recently by Van Der Weyden [17]. While a considerable boost in funding occurred in 2008 with the NHMRC package supporting a program for capacity-building grants for health services research, he notes that this funding has been made available in the absence of a comprehensive research agenda which is aligned to the health reform process.

Connelly et al [13] identify a lack of connection between health policy initiatives and existing strategies in place to achieve such initiatives. An emphasis on inter-sectoral action, inter-professional learning and working to a shared agenda is not supported by the human resource planning necessary to achieve these outcomes. Nor are the links to education and training clear. These authors recommend a 'joined up' human resources plan. However any attempt to align policy initiatives and implementation at the organisational level should acknowledge the growing 'peoples voice' [18]. There is increasing support for the 'people principle' through citizen juries and 'wrap around services' which refers to a more individualised and community approach to service delivery [19]. Mooney's evidence suggests that citizens are more supportive of public health and preventive medicine; an essential element for the proposed health reform processes.

Conway et al [14] note that while the term 'workforce development' is increasingly popular in the health care field there is little evidence to support a systems approach. These authors note an absence of systems and processes which would facilitate overall integration between organisational goals, human resource management policies and education and training, within an evaluated framework. A conceptual framework should distinguish the dynamic interaction of people, systems and processes working in a contextual situation and acknowledging the synergistic impacts of input, processes and outcomes.

A systems approach to public health workforce development has been previously associated with significant major organisational development and redesign in the public health sector [15]. Kennedy and Moore argue that developing organisations capable of knowledge creation may be the greatest determinant of how public health agencies perform in the 21^st ^century. Even greater challenges will arise with an increasing focus on prevention within the Australian health care system and the anticipated reorientating of services.

A report from the Australian Primary Health Care Research Institute [4] argues that organisational development has the potential to improve the effectiveness of the primary health care workforce in the future. The report recognises the components of leadership, culture and inter-professional collaboration as being essential to delivering better performance within an organisational development framework. It identifies organisational development as having the potential to contribute answers to issues related to chronic disease management, workforce supply and integration of care across organisational boundaries. The recognition and value of a social and behavioural science model in the primary health care sector [20] provides a tool to monitor improved organisational performance but the report does not expand on the inclusion of broader public health interventions across the continuum of care, or how inter-disciplinary workforce development would be included.

A workforce development framework for the public health nutrition workforce has been proposed by Hughes [21]. This framework involves an analysis of workforce development categories which include building human resource infrastructure (quantity), organisational systems and policy (quality), intelligence support (performance), learning systems (quality) and workforce preparation. However, he notes that this framework will require service reorientation and a level of unprecedented collaboration between academic, industry and community sectors. If effective strategies are to be adopted to address prevention across the continuum of care such issues would be expected to arise, with increased engagement between the public health and industry partners, particularly at the primary health care interface.

The New Zealand Ministry of Health, Public Health Workforce Development Plan 2007–2016, outlines a number of actions to address wider public health workforce development [22]. The plan adopts a systems approach that acknowledges a broad view of public health workforce development. It recognises the strategic importance of education and training for the broader health workforce including the primary health care sector, public clinical training programs and those working in community-based organisations. Investments will be made to provide support and input to professional training and competency development for public health/health promotion skills for the wider workforce. Work is being undertaken to integrate primary health care and public health training qualification frameworks and strengthen alignment across sectors.

Another example of workforce development aligned to key priorities is demonstrated in the NHS London Board, 'Workforce for London A Strategic Framework' released in September 2008 [23]. Goals in this framework support a major shift of medical and nursing staff from the acute to the community settings. Other goals include aligning workforce planning with service needs and a significant increase in investment to support continuing professional development for the existing workforce which is aligned to service needs. The strategy is underpinned by targets for London education providers to improve quality ratings.

The World Health Organization has recognised the importance of broad multi-sectoral approaches in various settings and the importance of public health and clinical collaboration in the prevention of some of the most serious global health challenges. For example, the Global School Health Initiative [24] has fostered approaches to prevent Helminth infection in 1996 and HIV/STI infection in 1997, in China. Such initiatives while predicated upon partnership and collaboration have not, however, led to any global programs to join up policy and health workforce development.

In 2008 the Scottish Government released a report examining the evidence related to a shift in the balance of care in health and community care with the aim of identifying the contribution of service delivery changes intended to improve health outcomes. The three areas examined were the shifting focus of care, shifting location of care and changing roles and responsibilities of patients and professionals [25]. While there were high levels of evidence analysing the shift in focus of care for the elderly, chronic disease and rehabilitation; significant research gaps were found in relation to shifting the focus of care for preventive and assessment-based interventions. There is limited evidence to support a shift to more extended primary and community care team approach supporting the potential for a range of roles to be developed or substituted, including those of nurses, allied health and lay workers in primary care. There was a small body of high level evidence to support the shift of responsibility to patients through greater use of technology and self management education.

In the Australian context, there has been little research to investigate a systematic partnership model between health policy makers, human resource professionals and the academic sector, to construct and support a comprehensive workforce development strategy, itself aligned to a more focused prevention agenda. There has been general agreement in the area of professional education and in the tertiary education sector that there are benefits from a multi-disciplinary approach. Thus, for example, many nursing education programs include aspects of health promotion in their coursework; also epidemiological and population-based coursework is often included in medical education programs; aspects of health service management and economic analysis models may be included in health professional programs. However, at a system wide level, new partnerships for public health and clinical practice in primary health care and community settings have not been identified, investigated and supported – and this needs to change.

Despite good evidence about returns on investment [26], public health has faced an enormous challenge in terms of its image. The nature and extent of 'Public Health' is poorly understood by the general public and is typically linked to the public hospital system. One explanation may be, as Lin and Robinson [27] argue, that:

*Public health is a small component of the health system, both in terms of budgetary allocation at either state or national level and in terms of the number of practitioners. It incorporates a myriad of activities; legislation and regulation for health protection, preventive services directed at specific diseases and populations, and health promotion programs geared towards particular risk factors and vulnerable groups in the community. As such, it looks like a disparate collection of programs and investments*.

The multi-disciplinary nature of public health may also have contributed to its relative isolation from interaction with the broader health system. Public health has few models for health service delivery, with accompanying funding models, in the acute or primary health care sector. However, even without such models, public health has the conceptual capacity required to address viable and sustainable approaches to the prevention agenda in an efficient and effective manner in the 21^st ^century.

While the public health lobby has secured a place in the spotlight for prevention and population approaches to chronic disease management in the current health reform agenda, it is still unclear whether system-wide change will occur; or merely 'tinkering' at the margins of existing service delivery, education and funding structures.

In summary, until recent developments, public health has failed to achieve significant political or broader recognition in terms of the financial and human returns on investment for the multiple prevention interventions in Australia. While it is not within the scope of this paper to resolve leadership, infrastructure and advocacy issues, or to propose new public health service delivery, workforce or funding solutions, clearly public health must act decisively to build on recent success in shaping reform and demonstrate actions and solutions which engage with the current preventive health agenda and engage more actively in workforce reform dialogue.

The NHHRC has briefed the National Preventative Health Task Force to provide advice to health providers and the government on evidence-based preventive programs to address the burden of chronic disease [10]. While acknowledging the complexity of this task, it is anticipated by many that a large proportion of this strategy will be directed towards the primary health care interface where to date, the workforce is comprised of clinically trained personnel.

The Taskforce discussion paper titled 'Australia: the healthiest country by 2020' [28] proposes a number of options for tackling the burden of chronic disease in Australia related to alcohol, tobacco and obesity. The paper acknowledges the importance of national leadership and coordination and proposes the establishment of a National Prevention Agency (NPA). Such an agency would support

"The coordination of partnerships and interventions, ensuring the relevance and quality of workforce training activities, social marketing, public education and the monitoring and evaluation of interventions"

The taskforce acknowledges that any successful attempt to address prevention must include the integration of new strategies into the national infrastructure and not just short term projects, despite such being the history of public health funding in Australia. However, there continues to be an apparent absence of strategic policy alignment for workforce development or consideration for health services in research agendas.

The successful implementation of the Taskforce recommendations will include many challenges, including those for workforce development. Such challenges, though not new, will gain added significance as the clinical workforce intersects with a population health paradigm and seeks to extend prevention beyond previous boundaries. Appropriate models to address these challenges have yet to be tested. Many authors have identified the link between an adequately skilled workforce and health system performance [12-15] and [16]. Therefore, during the current climate of reform, the identification of appropriate systems and processes for health workforce development between these two sectors, both aligned to the national preventive agenda, becomes more significant.

Allengrante et al [29] identify critical competencies for the public health education workforce in line with a changing health agenda which include coalition building, strategic planning, community health development, advocacy, business management, leadership and cultural competence. Allengrante's study identifies particular commonalities for workforce development relevant between academic and industry sectors and notes that any successful integration of public health competencies into continuing education will require cooperation from a broad range of groups including professional associations, universities and government and non-government sectors. Similarly, we argue that the intersection of the clinical and public health paradigms will demand comparable cooperation and shared agendas across sectors.

The move to a preventive agenda highlights the importance of collaborative workforce partnerships between the primary health care, public health and education sectors to facilitate the planning and implementation of more broadly based and evaluated inter-sectoral and multi-disciplinary education. By adopting Connelly et al's [13] vehicle for new forms of collaborative workforce development through 'joined up plans', new systems and processes for workforce development aligned to preventive health policy and organisational development, and an integrated plan for continuing professional development for primary health care should transpire.

A national coordinated response to the preventive agenda for the health workforce which is supported financially would contribute to the consistency, sustainability and quality of evidence-based continuing professional development. Resources to support effective partnerships and collaborative planning between education providers and relevant health care organisations would ensure continuing professional development is consistent and linked to research, quality and evidence. Quality frameworks for public health education currently exist in the tertiary and vocational sectors which could provide the foundation for the development of appropriate training courses for a broader health workforce in the primary health care setting [30,31]. Further evaluation of these competencies will need to occur in line with policy reforms and the proposed core health workforce competency framework currently being investigated by the National Health Workforce Taskforce [10].

Universities offer formal awards for public health from undergraduate to postgraduate levels, along with various forms of continuing professional education in the form of short courses, workshops and seminars. This contribution to workforce capacity building can be considered at three levels of skill: generic skills, specialised skills and high profile specific specialist skills in areas of specific strategic need. The Population Health Training Package through the TAFE sector provides education from a Certificate II to Diploma level in Population Health which covers vocationally orientated population health training. The appropriate design and delivery of future public health education for the broader workforce should be closely aligned to these quality frameworks with identified competencies; aligned to overarching policy initiatives, resourcing and rigorous evaluation of workforce development outcomes.

## Conclusion

This paper considers the potential implications for health workforce development as a result of the NHHRC preventive agenda; particularly in reference to primary health care and public health. The rationale for effective partnerships, aligned agendas and supportive resources for workforce development are discussed. Issues related to human resource development within an organisational context are raised and the need to consider a nationally consistent approach to workforce development and quality continuing professional development is discussed.

As noted, public health has a minor budgetary allocation with limited numbers of identified workforce within the Australian health system. In the absence of any predicted revolutionary changes, larger sections of the health workforce will be required to adopt population health models and values to transform preventive health care beyond existing perspectives and models. Through effective and resourced partnerships new models of primary care and brief public health interventions, supported by evidence, will change the structure and delivery of primary health care and impact positively on decreasing the burden of preventable disease in the Australian population.

This paper argues that in order to achieve an effective response to the preventive agenda, human resources for health require improved recognition and alignment to policy initiatives. New clinical and public health models, education, infrastructure, an aligned research agenda and sustainable resourcing will all be essential elements of the integrated response.

## Competing interests

The authors declare that they have no competing interests.

## Authors' contributions

KCL developed the paper with input from DES. Both authors read and approved the second draft.
